# Plasmonic Sensors for Monitoring Biological and Chemical Threat Agents

**DOI:** 10.3390/bios10100142

**Published:** 2020-10-15

**Authors:** Yeşeren Saylan, Semra Akgönüllü, Adil Denizli

**Affiliations:** Department of Chemistry, Hacettepe University, 06800 Ankara, Turkey; yeseren@hacettepe.edu.tr (Y.S.); semraakgonullu@hacettepe.edu.tr (S.A.)

**Keywords:** biological threat agents, chemical threat agents, monitoring, plasmonic sensors

## Abstract

Sensors are excellent options owing to their ability to figure out a large number of problems and challenges in several areas, including homeland security, defense, medicine, pharmacology, industry, environment, agriculture, food safety, and so on. Plasmonic sensors are used as detection devices that have important properties, such as rapid recognition, real-time analysis, no need labels, sensitive and selective sensing, portability, and, more importantly, simplicity in identifying target analytes. This review summarizes the state-of-art molecular recognition of biological and chemical threat agents. For this purpose, the principle of the plasmonic sensor is briefly explained and then the use of plasmonic sensors in the monitoring of a broad range of biological and chemical threat agents is extensively discussed with different types of threats according to the latest literature. A conclusion and future perspectives are added at the end of the review.

## 1. Introduction

Two major threat agents are utilized in attacks—biological and chemical threat agents—and the detection of these agents is a modern subject of increasing impact and interest [1]. The Nobel Peace Prize awarded to the Organization for the Prohibition of Chemical Weapons in 2013 “for its extensive efforts to eliminate chemical weapons” sheds light on modern-age issues such as this [2]. Bioterrorism is a deliberate use of harmful biological threats agents that provoke disease or death in plants, animals, and humans [3]. Unfortunately, these dangerous agents can spread through the air, water, or soil [4]. Moreover, they can be modified to increase their disease-causing properties that make them resistant to existing drugs and improve their ability to spread to the environment [5,6]. Compared to chemical threat agents, biological threat agent’s production is much cheaper and also the danger area and expected loss of life is more effective than chemical threat agents in a military attack [7]. The infection dose—the amount of organism required for an infection outbreak—is different for each threat agent [8]. The risk ratio of each threat agent is given not only by infection dose, but also by the natural propagation pathway, aerosol or water stability, and the possibility of spore formation in the case of bacteria [9]. In particular, biological threat agents obtain toxic substances that are relatively easy and inexpensive, and they can easily spread and cause fear and panic beyond real physical damage [10,11].

Several analytical techniques have been used to detect the various biological and chemical threat agents over the years [12,13,14,15,16,17,18,19,20]. Researchers have been using analytical technique combinations including extraction, mass spectroscopy, liquid chromatography, and sensors in numerous situations due to the different needs that are hard to detect into a single suitable monitoring system and methodology [21,22,23,24,25,26]. There is a need for the development of novel sensing methods with smart detection capabilities in defense and homeland security applications for the early detection of biological and chemical threat agents. A useful and commercially attractive sensor systems for accurate and rapid detection of biological and chemical threat agents has to satisfy some important properties [27]. Various recent studies indicate that plasmonic sensors can be a key platform for monitoring biological and chemical threat agents owing to their combination of different charming properties such as sensitivity, rapid, unlabeled, low cost, real time, and portability [28,29,30,31,32,33,34,35,36,37,38].

On the other hand, despite a fast-growing quantity of articles, there is no up-to-date review of plasmonic sensors for monitoring biological and chemical threat agents. This review purposes to fill this gap by summing up the most recent advances in this subject. Following an introduction to the principle of sensors and plasmonic sensors (Section 2), the importance of the monitoring of these dangerous agents (Section 3). Then, a wide overview is discussed about the performance and characteristics of published plasmonic sensors for the detection of biological (Section 3.1) and chemical (Section 3.2) threat agents. On the whole, it was hoped that this review article will conduct and inspire all researchers for further plasmonic sensors developments and applications.

## 2. Principle of Sensors

**Figure 1:**
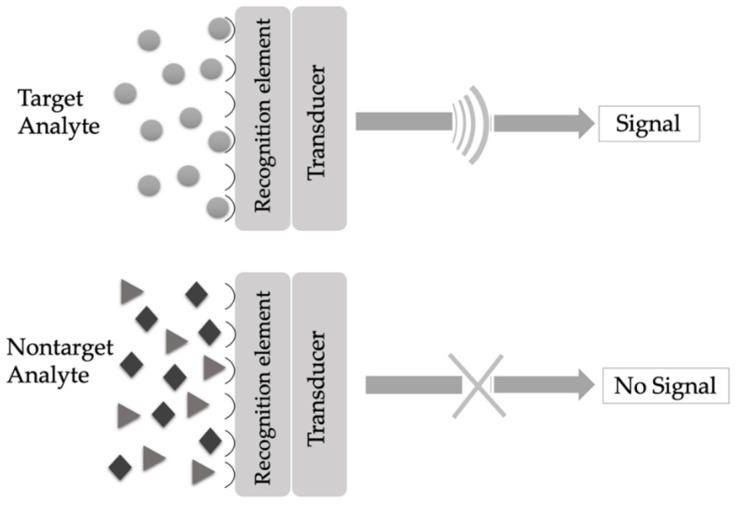
Working principle of a sensor.

A sensor is an analytical tool that has three main modules: a sensing receptor, a transducer, and a detector [39]. As demonstrated in Figure 1, a target analyte principally binds to the recognition element (receptor), and the sensing component specifically recognizes the analyte by a reaction, specific adsorption, or another process as physical and/or chemical and then the transducer translates changes to a quantifiable signal measured by the digital detector module [40].

**Figure 2:**
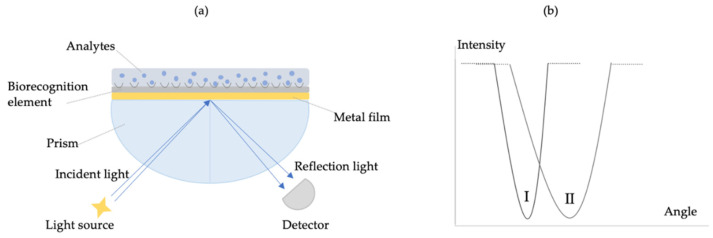
Scheme of plasmonic sensor with a metal and recognition layer (**a**) and change in the signal due to the increase of angle in the proximity of a sensor surface induced by captured target analytes (**b**).

Sensors have numerous advantages, including user-friendly operation, exceptional performance, rapid response, minimum sample preparation and processing, portability, high sensitivity and specificity, relatively compact size, and real-time analysis that provide many advantages compared to standard analytical methods [41,42,43,44,45]. Furthermore, they have a great impact on the environment, medical, safety, and many other applications [46,47,48]. Detection elements are very diverse. They include conductivity, density, an electromagnetic radiation phase, electric current, mass, viscosity, electrical potential, temperature, and impedance [49]. In recent years, plasmonic sensors have attracted broad attention due to their perfect electromagnetic control ability. Owing to the physical and optical properties of the plasmonic sensors, they have become the most famous device for working [50]. These unique properties make them an indispensable real-time and label-free platform for target analyte detection. The current focus of the field is to further be growing the sensitivity to detect low concentrations or even single molecules in diluted solutions [51]. So, they are promising for ultrasensitive detection and have received increasing applications in biological and chemical analysis, food control, clinical diagnostics, biomedical research, etc. [52]. Plasmonic sensors also focus on the analysis of a change of intensity in the optical characteristics of the transducer metal surface when the target analyte is captured by the recognition element (Figure 2). They are divided into many subclasses such as resonance, fluorescence, refraction, reflection, dispersion, phosphorescence, infrared absorption, Raman scattering, and chemiluminescence [53,54,55].

Plasmonic sensors belong to the group of optical-based affinity sensors. When the target analyte is captured by the ligand, it causes a measurable signal. Various sensor surfaces with immobilized ligands are commercially available and/or can be custom designed and fabricated [56]. In general, plasmonic sensors are created of sensing elements that contain metal or metal-dielectric nanostructures supporting surface plasmons and recognition elements that can selectively bind a target analyte. The enlightenment of the sensing element by light produces surface plasmons on the structures generating the electromagnetic field that is highly concentrated at the surface of the structures. When a sample containing target analyte is brought into contact with the sensor, the capture of the target analyte by recognition element immobilized on the surface of the sensing element gives rise to a change in the refractive index in the region close to the surface [57]. The surface plasmons are electron oscillations that take place at the limit of a noble metal (plasmonic materials) and different media as an aqueous solution (dielectric material). Changes in the dielectric constant, or refractive index, near the surface of the plasmonic sensor, are measured as a change in the angle or wavelength of p-polarized light absorbed by the surface plasmon. The metal film surface, usually gold or silver, is modified with surface chemistry steps to immobilize a series of molecular recognition elements. The molecular recognition element is used to capture the specific binding partners [58,59,60].

## 3. Monitoring Biological and Chemical Threats Agents

Many researchers, including chemists, biologists, physicists, engineers, and medical doctors, have used the sensor platforms as an original application in different areas for the development of sensors [61,62,63,64,65]. Today, the early detection of a biological and chemical attack of the threats can only be analyzed with commercial methods. Thus, real-time sensors are needed for the security of the community [66]. The rapid and specific detection of threat agents is a critical aspect of defense. Monitoring systems must be fast, responsive, portable, and also specific to threat agents [67,68]. Interest in the quality of life is increasing all over the world. It is maintained by various factors, such as quality of life, disease control, drug development, environmental cleaning, food safety, and homeland security [69,70].

### 3.1. Biological Threats Agents

Biological threat agents are classified into three categories as depicted in Table 1. In Category A, the agents can easily spread or spread from person to person. This results in high death rates and has a large impact on common health. It can also cause social disruption and public anxiety and need a special solution for health preparation. In Category B, the agents are partially easy to spread. It results in a low disease rate and death and requires specific improved diagnostic capacity and disease surveillance. In Category C, the agent can be designed for mass spreading due to their future availability. It is easy to produce and spread. These are potentially linked to high disease and death rates and high health effects [71,72,73,74,75,76,77,78].

Sharma et al. screened three monoclonal antibodies (mAb1, mAb2, and mAb3) of Ebola virus employing a surface plasmon resonance (SPR) sensor to pick a suitable antibody for against a biological threat agent [79]. They first modified a gold chip surface with 4-mercaptobenzoic acid and then immobilized with the recombinant nucleoprotein of the Ebola virus. They calculated the affinity constants as 809 nM, 350 pM, and 52 pM for mAb1, mAb2, and mAb3 of the Ebola virus interaction, respectively. They concluded the high affinity of mAb3 with the Ebola virus and confirmed this result with ELISA. Finally, the limit of the detection value of the plasmonic sensor is calculated as 0.5 pg/mL for mAb3. 

**Figure 3:**
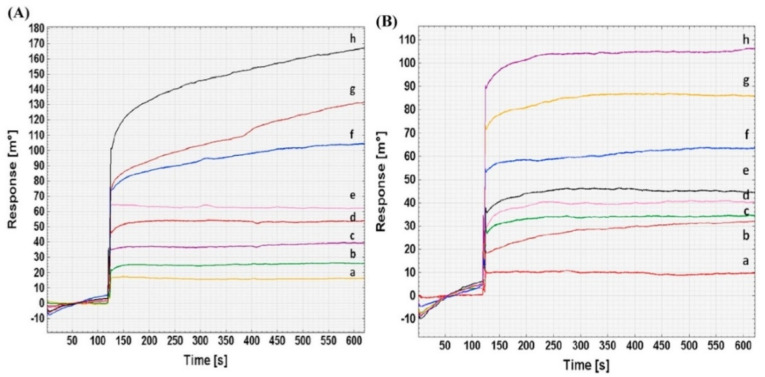
Surface plasmon resonance (SPR) sensor responses for different dilutions’ (1:12,800 (a), 1:6400 (b), 1:3200 (c), 1:1600 (d), 1:800 (e), 1:400 (f), 1:200 (g) and 1:100 (h)) interaction with the DNA target for Probe 1 (**A**) and 2 (**B**). Republished with permission from [80]; permission conveyed through Copyright Clearance Center, Inc.

Sikarwar et al. prepared an SPR sensor using 4-mercaptobenzoic acid-modified gold for *Brucella melitensis* detection using its complementary DNA targets with two different probes of the IS711 gene [80]. They performed kinetics and thermodynamic analysis, and the results reflected that complementary DNA targets and Probe 1 are a more effective interaction than Probe 2 (Figure 3). Furthermore, they carried out the real serum samples’ analysis and reported the applicability of this plasmonic sensor for *Brucella melitensis* detection in less than 10 min.

Patel et al. provided an SPR sensor for Botulinum Neurotoxin type A Light Chain detection to show the feasibility of the Newton Photonics-based sensor [81]. The limit of detection was calculated as 6.76 pg/mL and they established that the detection sensitivity of the plasmonic sensor is comparable to the traditional mouse LD50 bioassay.

**Figure 4:**
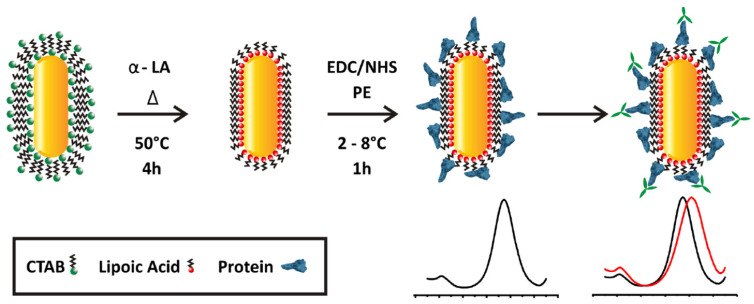
Modification steps of Dengue virus localized surface plasmon resonance (LSPR) sensor surface. Republished with permission from [82]; permission conveyed through Copyright Clearance Center, Inc.

Versiani et al. developed a gold nanorod functionalized plasmonic sensor based on localized surface plasmon resonance (LSPR) for Dengue virus protein detection [82]. They first linked the gold nanorods to α-lipoic acid (α-LA) by interaction with the thiol group and then 1-ethyl-3-(3-dimethyl aminopropyl) carbodiimide (EDC) and N-hydroxysuccinimide (NHS) are mixed to stabilize substitution reactions owing to recombinant proteins covalently bound to α-LA on the gold nanorod surfaces. They used this protein-decorated gold nanorod as a sensor that shifts the emitted spectrum (Figure 4). They reported that this plasmonic sensor can detect one picogram of anti-Dengue virus monoclonal antibodies in Dengue virus-positive human sera. They also performed the cross-selectivity experiments with Zika-virus-infected patients and showed that this sensor can distinguish Dengue virus serotype infected individual patients.

Surface-enhanced Raman spectroscopy (SERS) depends on plasmonic platforms and nano-antenna, which have developed into an effective area of study [83]. SERS is one of the leading techniques for label-free ultrasensitive vibrational fingerprinting of a variety of compounds. SERS has been identified as key platform thanks to distinctive features such as: ultrahigh sensitivity, detection from a wide variety of matrices and quantification of multiple species in a single measurement, allowing for real-time detection in the field [84].

**Figure 5:**
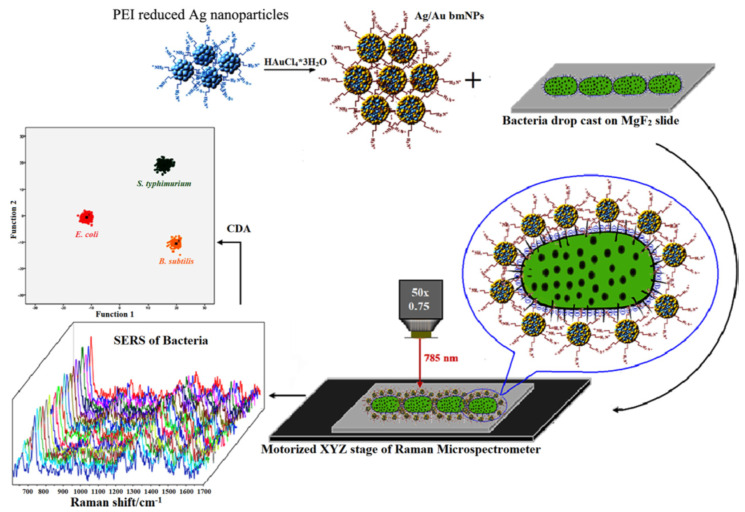
Scheme of a surface-enhanced Raman spectroscopy (SERS)-based sensor for bacteria detection. Republished with permission from [85]; permission conveyed through Copyright Clearance Center, Inc.

Prakash et al. demonstrated positively charged silver/gold bimetallic nanoparticles for *Escherichia coli*, *Salmonella typhimurium*, and *Bacillus subtilis* detection using surface-enhanced Raman scattering (SERS)-based sensor [85]. This plasmonic sensor has important advantages, such as high sensitivity, short detection time at low power, and an easy operating process. They obtained a reusable and specific cell wall fingerprint and intracellular components of these bacteria by SERS that allows for the differentiation and classification of these bacteria employing multivariate analyses (Figure 5).

**Figure 6:**
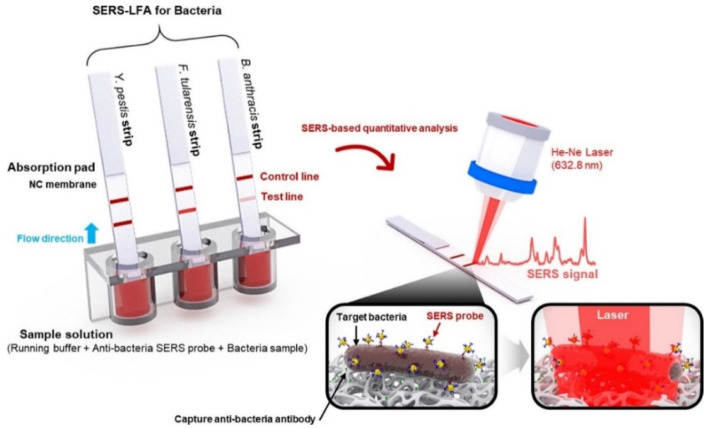
Principle of SERS-based sensor for bacteria detection. Republished with permission from [86]; permission conveyed through Copyright Clearance Center, Inc.

Wang et al. also built up an SERS-based sensor for bacterial pathogen (*Francisella tularensis*, *Bacillus anthracis*, and *Yersinia pestis*) detection [86]. They first dipped the lateral flow assay (LFA) strips into well-plates containing mixtures of SERS nanotags (Raman reporter-labeled gold nanoparticles) and broad concentrations of bacteria in buffer solution and then formed complexes that relocated by capillary action to measure and analyze Raman signals (Figure 6). They needed a short time (15 min) and a low volume (40 μL) to obtain this highly selective detection. 

### 3.2. Chemical Threat Agents

Chemical threat agents are inactive molecules that are classified into several categories in terms of the effects on the human body. Even though their usage is forbidden, they are still employed for dirty aims [87]. The chemical threat agents are generally separated as the following classes: nerve agents, blood and suffocating agents, vesicant and blister agents, cytotoxic proteins, pulmonary agents, incapacitating agents, and lachrymatory agent. The first three are best known according to their high toxicity, and the researcher also utilizes their mimics for investigation (Table 2). So, the rapid monitoring of the chemical threat agents is highly necessary and urgent [88,89,90,91,92].

**Figure 7:**
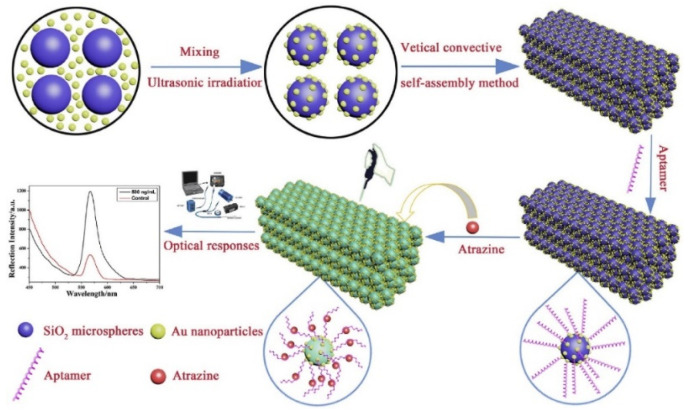
Scheme of preparation photonic crystal-based sensor for atrazine detection. Republished with permission from [93]; permission conveyed through Copyright Clearance Center, Inc.

Song et al. investigated an LSPR-based signal enhancement mechanism using photonic crystals sensing material that was modified with silica microspheres and gold nanoparticles for atrazine detection [93]. They first modified the surface of silica microspheres with gold nanoparticles and then immobilized with atrazine aptamer (containing 5′-terminal mercapto group) for specific atrazine detection with the intensity change of the photonic crystals (Figure 7). They easily prepared this LSPR-based photonic crystal and reached ultrasensitive detection (10^−12^ g/mL) in the 0.0001 ng/mL to 500 ng/mL range without any label.

**Figure 8:**
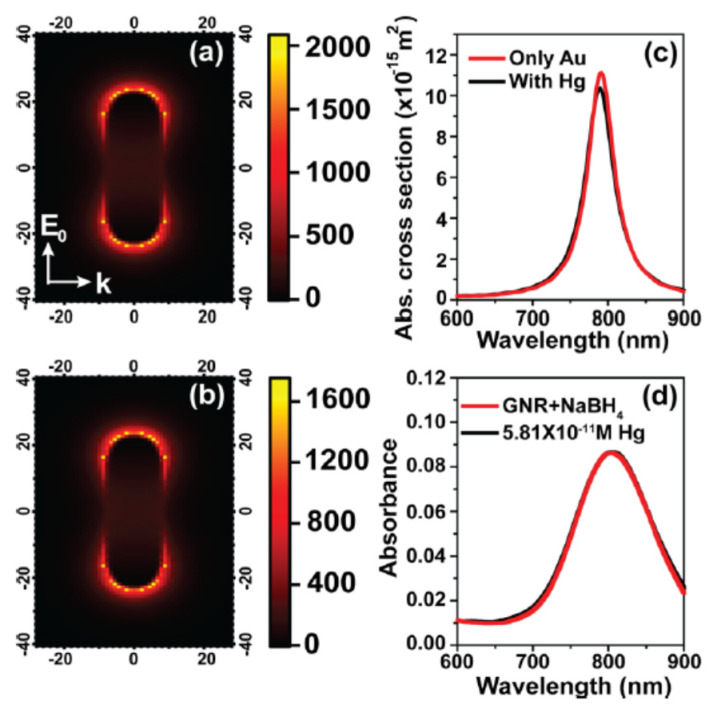
Electric field enhancement in the absence (**a**) and presence (**b**) of mercury; simulated (**c**) and experimental LSPR spectra (**d**) for comparison. Republished with permission from [94]; permission conveyed through Copyright Clearance Center, Inc.

Verma and Chandra presented a nonlinear plasmonic sensor that depended on nonlinear optics combination with amalgamation chemistry and plasmonic properties of gold nanorods for mercury detection [94]. They prospered an improved limit of detection value of mercury as compared to LSPR-based sensing by operating with ultra-high sensitivity in the nonlinear optics process against mercury-induced change in the plasmonic nanorod’s local electric field (Figure 8) and achieved as low as (58 pM) the limit of detection value with high selectivity. They also reported that the mercury amount utilized in the simulation corresponded to a concentration of 10^−11^ M.

**Figure 9:**
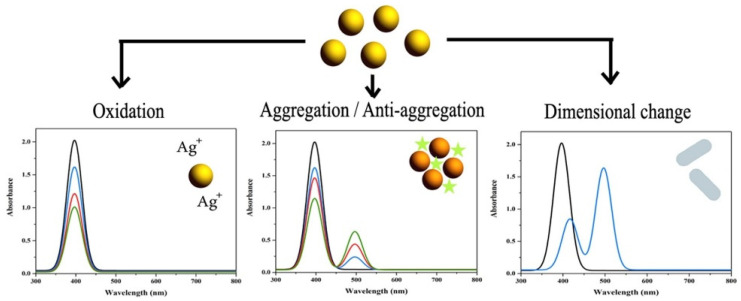
Sensing mechanisms of gold nanoparticle-based plasmonic sensors. Republished with permission from [95]; permission conveyed through Copyright Clearance Center, Inc.

Amirjani and Haghshenas summed up recent studies about silver nanoparticle-based plasmonic sensors for heavy metal ion (Co^2+^, Hg^2+^, Cd^2+^, Pb^2+^, and Cu^2+^) detection [95]. As depicted in Figure 9, they dared to shed more light on the monitoring of LSPR properties of silver nanoparticles that depended on the changes of aggregation, anti-aggregation, oxidation reduction, and dimensional-morphological. They mentioned that the cost-effective and excellent plasmonic feature of silver compared to gold stems from its imaginary part of the dielectric function which is close to zero in a wide range of wavelengths.

**Figure 10:**
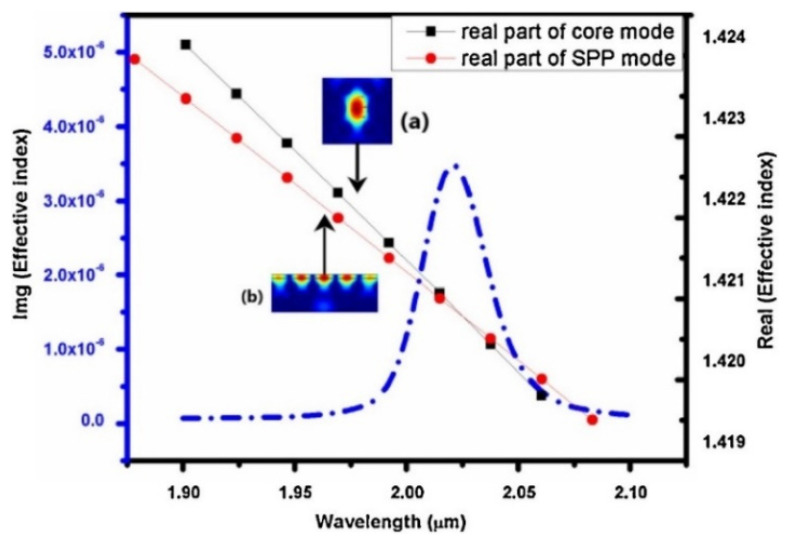
Coupling of silica (black dotted line), SPP (red dotted line), and the imaginary part of silica (blue dotted line) modes of maximum wavelength. Republished with permission from [96]; permission conveyed through Copyright Clearance Center, Inc.

Thenmozhi et al. advanced an SPR sensor using the Finite element method for 1, 4-dioxane, and diethanolamine detection [96]. They first coated the sensor’s surface with indium titanium oxide and placed it over the analyte, which increased the plasmon excitation on the surface. As shown in Figure 10, the coupling of silica, surface plasmon polariton (SPP), and the imaginary part of silica modes of maximum wavelengths have the energy distribution of silica and SPP modes, respectively. Then, they succeeded in the maximal spectral sensitivity (50,000 nm/RIU) participated with high-resolution (4 × 10^−4^ RIU) and also maximum amplitude sensitivity (1266.67 RIU^−1^). 

Heleg-Shabtai et al. developed the SERS-based sensor made by hand-held Raman spectrometers for gas-phase nerve (methylphosphonothioic acid: VX) and blister (sulfur mustard: HD) agents monitoring [97]. They comprised the sensor with gold nanoparticles that were modified onto quartz fibers and performed gas-phase experiments utilizing a homemade flow system. They also optimized different SERS methods for VX and HD detection in solution and calculated the limit of detection values as 1.8 × 10^−3^ μg/mL and 2.5 × 10^−3^ μg/mL for HD and VX, respectively. 

**Figure 11:**
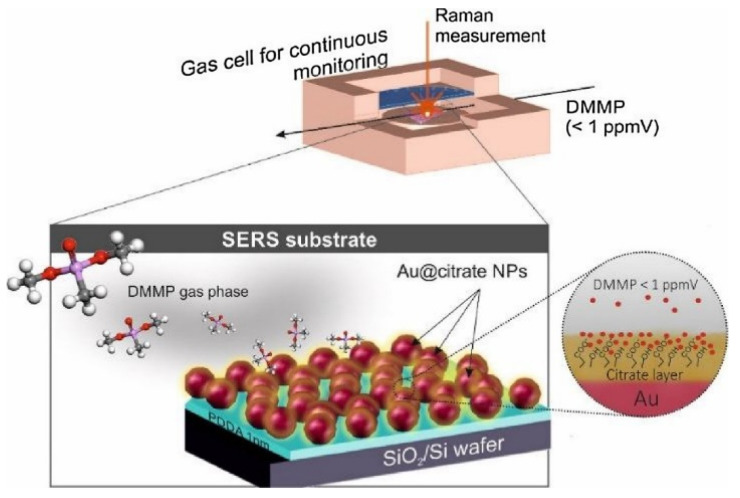
Interaction of dimethyl methyl phosphonate (DMMP) with an SERS-based sensor. Republished with permission from [98]; permission conveyed through Copyright Clearance Center, Inc.

Lafuente et al. also prepared the SERS-based sensor for gas-phase monitoring of dimethyl methyl phosphonate (DMMP) that is a surrogate molecule of nerve agents [98]. They designed the plasmonic sensor using gold nanoparticles that coated with a citrate layer that reacts as a powerful trap of the DMMP by hydrogen bonding interactions under optimum conditions (Figure 11). They reported that this plasmonic sensor can monitor low concentrations of DMMP (130 parts-per-billion) in the gas phase.

## 4. Conclusions and Future Perspectives

Defense against the threats is based on the early monitoring of the biological and chemical agents, the separation of infected individuals, and the assessment of the contaminated area. Therefore, fast, sensitive, and portable platforms are required for the real-time detection of these threats. Plasmonic sensor-based platforms have become an important part of laboratories. These sensors provide similar sensitivity compared to other conventional platforms. Owing to their small size and low cost, plasmonic sensors are suitable not only in the laboratory routine but also for mobile laboratories and field-portable systems. Therefore, there is a clear need to develop new technologies to act immediately and detect long-term threat agents in the event of the release of both intentional and unintentional agents. In this review, the recent developments of plasmonic sensors are overviewed for biological and chemical threat agents’ detection. Compared with conventional platforms that often need labor-intensive sample preparation and advanced instrument, these platforms demonstrate more encouraging applications for human health and life improvement. Even though there are many solutions to detecting biological and chemical threat agents, there is still considerable room for improvement. Understanding the mechanisms of how the biological and chemical threat agents attack and destroy the living is obligatory to create new strategies for monitoring, discriminating, and destroying these agents. The growth of plasmonic sensors for many of these agents would be a step towards capabilities. 

## Figures and Tables

**Table 1:** Main biological threat agents [78].

Group	Diseases	Agents
**A**	Anthrax	*Bacillus anthracis*
	Botulism	*Clostridium botulinum toxin*
	Plague	*Yersinia pestis*
	Smallpox	*Variola major*
	Tularemia	*Francisella tularensis*
	Viral hemorrhagic fevers	*Filoviruses and Arenaviruses*
**B**	Brucellosis	*Brucella spp.*
	Epsilon toxin	*Clostridium perfringens*
	Food safety threats	*Salmonella spp., E.coli O157:H7, Shigella*
	Glanders	*Burkholderia mallei*
	Melioidosis	*Burkholderia pseudomallei*
	Psittacosis	*Chlamydia psittaci*
	Q fever	*Coxiella burnetii*
	Ricin toxin	*Ricinus communis*
	Staphylococcal enterotoxin	*Staphylococcus spp.*
	Typhus fever	*Rickettsia prowazekii*
	Viral encephalitis	*Alphaviruses*
	Water safety threats	*Vibrio cholera, Cryptosporidium parvum*
**C**	Emerging infectious diseases	*Nipahvirus and Hantavirus*

**Table 2:** Main chemical threat agents. Republished with permission from [87]; permission conveyed through Copyright Clearance Center, Inc.

Group	Agent	Action Mode
**Nerve agents**	G-group (Tabun, soman, sarin) V-group (VX, Vx, CVx, VR) A-group (Novichoks)	Inactivates the enzyme acetylcholinesterase, preventing the breakdown of the neurotransmitter acetylcholine in the victim’s synapses and causing both muscarinic and nicotinic effects.
**Vesicant/Blister**	Sulfur mustard Nitrogen mustard	Agents are acid-forming compounds that damage skin and respiratory system, resulting in burns and respiratory problems.
**Blood/suffocating**	Cyanogen chloride Hydrogen cyanide	Cyanide directly prevents cells from using oxygen. The cells then use anaerobic respiration, creating excess lactic acid and metabolic acidosis.

## References

[1] Hakonen A., Andersson P.O., Schmidt M.S., Rindzevicius T., Kall M. (2015). Explosive and chemical threat detection by surface-enhanced Raman scattering: A review. Anal. Chim. Acta.

[2] Organisation for the Prohibition of Chemical Weapons. https://www.nobelprize.org/prizes/peace/2013/opcw/facts/.

[3] Green M.S., LeDuc M., Cohen D., Rfranz D. (2019). Confronting the threat of bioterrorism: Realities, challenges, and defensive strategies. Lancet Infect. Dis..

[4] Yadav I.C., Devi N.L., Syed J.H., Cheng Z., Li J., Zhang G., Jones K.C. (2015). Current status of persistent organic pesticides residues in air, water, and soil, and their possible effect on neighboring countries: A comprehensive review of India. Sci. Total Environ..

[5] Kirsch J., Siltanen C., Zhou Q., Revzin A., Simonian A. (2013). Biosensor technology: Recent advances in threat agent detection and medicine. Chem. Soc. Rev..

[6] Anzai J.I. (2015). Chapter 62: Biosensors for the Detection of OP Nerve Agents. Handbook of Toxicology of Chemical Warfare Agents.

[7] Pohanka M., Skládal M., Kroèa M. (2007). Biosensors for biological warfare agent detection. Def. Sci. J..

[8] Walper S.A., Aragonés G.L., Sapsford K.E., Brown C.W., Rowland C.E., Breger J.C., Medintz I.L. (2018). Detecting biothreat agents: From current diagnostics to developing sensor technologies. ACS Sens..

[9] Sapsford K.E., Bradburne C., Delehanty J.B., Medintz I.L. (2008). Sensors for detecting biological agents. Mater. Today.

[10] Mondal B., Bhavanashri N., Mounika S.P., Tuteja D., Tandi K., Soniya H. (2020). Chapter 6: Microfluidics application for detection of biological warfare agents. Handbook on Biological Warfare Preparedness.

[11] Courtney B., Bond K.C., Maher C. (2014). Regulatory underpinnings of global health security: FDA’s roles in preventing, detecting, and responding to global health threats. Biosecur. Bioterror..

[12] Öztürk B.Ö., Şehitoğlu S.K. (2019). Pyrene substituted amphiphilic ROMP polymers as nano-sized fluorescence sensors for detection of TNT in water. Polymer.

[13] Guo C.X., Lei Y., Li C.M. (2011). Porphyrin functionalized graphene for sensitive electrochemical detection of ultratrace explosives. Electroanalysis.

[14] Douglas T.A., Walsh M.E., Weiss C.A., McGrath C.J., Trainor T.P. (2012). Desorption and transformation of nitroaromatic (TNT) and nitramine (RDX and HMX) explosive residues on detonated pure mineral phases. Water Air Soil Pollut..

[15] Xin Y.H., Wang Q., Liu T., Wang L., Li J., Fang Y. (2012). A portable and autonomous multichannel fluorescence detector for on-line and in situ explosive detection in aqueous phase. Lab Chip.

[16] Pesavento M., D’Agostino G., Alberti G., Biesuz R., Merli D. (2013). Voltammetric platform for detection of 2,4,6- trinitrotoluene based on a molecularly imprinted polymer. Anal. Bioanal. Chem..

[17] Leppert J., Horner G., Rietz F., Ringer J., Lammers P.S., Boeker P. (2012). Near real time detection of hazardous airborne substances. Talanta.

[18] Kartha K.K., Sandeep A., Nair V.C., Takeuchi M., Ajayaghosh A. (2014). A carbazole-fluorene molecular hybrid for quantitative detection of TNT using a combined fluorescence and quartz crystal microbalance method. Phys. Chem. Chem. Phys..

[19] Jang Y.J., Tsay O.G., Murale D.P., Jeong J.A., Segev A., Churchill D.G. (2014). Novel and selective detection of Tabun mimics. Chem. Commun..

[20] de Grenu B.D., Moreno D., Torroba T., Berg A., Gunnars J., Nilsson T., Nyman R., Persson M., Pettersson J., Eklind I. (2014). Fluorescent discrimination between traces of chemical warfare agents and their mimics. J. Am. Chem. Soc..

[21] Rodriguez N.M., Linnes J.C., Fan A., Ellenson C.K., Pollock N.R., Klapperich C.M. (2015). Paper-based RNA extraction, in situ isothermal amplification, and lateral flow detection for low cost, rapid diagnosis of influenza A (H1N1) from clinical specimens. Anal. Chem..

[22] Ho Y.P., Reddy P.M. (2010). Identification of pathogens by mass spectrometry. Clin. Chem..

[23] Franciosa G., Pourshaban M., De Luca A., Buccino A., Dallapiccola B., Aureli P. (2004). Identification of type A, B, E, and F botulinum neurotoxin genes and of botulinum neurotoxigenic clostridia by denaturing high-performance liquid chromatography. Appl. Environ. Microbiol..

[24] Fischer N.O., Tarasow T.M., Tok J.B. (2007). Aptasensors for biosecurity applications. Curr. Opin. Chem. Biol..

[25] Saylan Y., Yılmaz F., Özgür E., Derazshamshir A., Bereli N., Yavuz H., Denizli A. (2018). Chapter 10: Surface Plasmon Resonance Sensors For Medical Diagnosis. Nanotechnology Characterization Tools for Biosensing and Medical Diagnosis.

[26] Ferrier D.C., Shaver M.P., Hands P.J. (2015). Micro-and nanostructure based oligonucleotide sensors. Biosens. Bioelectron..

[27] Petkovic K., Swallow A., Stewart R., Gao Y., Li S., Glenn F., Gotama J., Dell’Olio M., Best M., Doward J. (2019). An integrated portable multiplex microchip device for fingerprinting chemical warfare agents. Micromachines.

[28] Joo J., Yim C., Kwon D., Lee J., Shin H.H., Cha H.J., Jeon S. (2012). A facile and sensitive detection of pathogenic bacteria using magnetic nanoparticles and optical nanocrystal probes. Analyst.

[29] Liu T., Zhao Y., Zhang Z., Zhang P., Li J., Yang R., Yang C., Zhou L. (2014). A fiber optic biosensor for specific identification of dead *Escherichia coli* O157: H7. Sens. Actuators B Chem..

[30] Mukundan H., Kumar S., Price D.N., Ray S.M., Lee Y.J., Min S., Eum S., Kubicek-Sutherland J., Resnick J.M., Grace W.K. (2012). Rapid detection of *Mycobacterium tuberculosis* biomarkers in a sandwich immunoassay format using a waveguide-based optical biosensor. Tuberculosis.

[31] Ohk S.H., Bhunia A.K. (2013). Multiplex fiber optic biosensor for detection of *Listeria monocytogenes*, *Escherichia coli* O157: H7 and *Salmonella enterica* from ready-to-eat meat samples. Food Microbiol..

[32] Erdem Ö., Saylan Y., Cihangir N., Denizli A. (2019). Molecularly imprinted nanoparticles based plasmonic sensors for real-time *Enterococcus faecalis* detection. Biosens. Bioelectron..

[33] Al-Rekabi S.H., Kamil Y.M., Bakar M.H.A., Fen Y.W., Lim H.N., Kanagesan S. (2019). Hydrous ferric oxide-magnetite-reduced graphene oxide nanocomposite for optical detection of arsenic using surface plasmon resonance. Opt. Laser Technol..

[34] Cittadini M., Bersani M., Perrozzi F., Ottaviano L., Wlodarski W., Martucci A. (2013). Graphene oxide coupled with gold nanoparticles for localized surface plasmon resonance based gas sensor. Carbon N. Y..

[35] Yu W.W., White I.M. (2013). Inkjet-printed paper-based SERS dipsticks and swabs for trace chemical detection. Analyst.

[36] Rahtuvanoğlu A., Akgönüllü S., Karacan S., Denizli A. (2020). Biomimetic nanoparticles based surface plasmon resonance biosensors for histamine detection in foods. ChemistrySelect.

[37] Yılmaz E., Özgür E., Bereli N., Türkmen D., Denizli A. (2017). Plastic antibody based surface plasmon resonance nanosensors for selective atrazine detection. Mater. Sci. Eng. C.

[38] Wang J., Jiang C., Wang X., Wang L., Chen A., Hu J., Luo Z., Wang S., Gambhir S.S., Weiss S. (2016). Fabrication of an “ion-imprinting” dual-emission quantum dot nanohybrid for selective fluorescence turn-on and ratiometric detection of cadmium ions. Analyst.

[39] Saylan Y., Erdem Ö., Ünal S., Denizli A. (2019). An alternative medical diagnosis method: Biosensors for virus detection. Biosensors.

[40] Saylan Y., Akgönüllü S., Yavuz H., Ünal S., Denizli A. (2019). Molecularly imprinted polymer based sensors for medical applications. Sensors.

[41] Bhalla N., Pan Y., Yang Z., Payam A.F. (2020). Opportunities and challenges for biosensors and nanoscale analytical tools for pandemics: COVID-19. ACS Nano.

[42] Inci F., Karaaslan M.G., Mataji-Kojouri A., Shah P.A., Saylan Y., Zeng Y., Avadhani A., Sinclair R., Lau D.T.L., Demirci U. (2020). Enhancing the nanoplasmonic signal by a nanoparticle sandwiching strategy to detect viruses. Appl. Mater. Today.

[43] Forzato C., Vida V., Berti F. (2020). Biosensors and sensing systems for rapid analysis of phenolic compounds from plants: A comprehensive review. Biosensors.

[44] Özgür E., Saylan Y., Bereli N., Türkmen D., Denizli A. (2020). Molecularly imprinted polymer integrated plasmonic nanosensor for cocaine detection. J. Biomater. Sci. Polym. Ed..

[45] Ansari S., Masoum S. (2019). Molecularly imprinted polymers for capturing and sensing proteins: Current progress and future implications. Trends Anal. Chem..

[46] Saylan Y., Erdem Ö., Inci F., Denizli A. (2020). Advances in biomimetic systems for molecular recognition and biosensing. Biomimetics.

[47] Saylan Y., Denizli A. (2020). Chapter 33: Virus Detection Using Nanosensors. Nanosensors for Smart Cities.

[48] Saylan Y., Erdem Ö., Yılmaz F., Cihangir N., Denizli A. (2018). Chapter 3: Sensor Application For Environmental Pollutants. Affinity Sensors.

[49] Farka Z., Juřík T., Kovář D., Trnková L., Skládal P. (2017). Nanoparticle-based immunochemical biosensors and assays: Recent advances and challenges. Chem. Rev..

[50] Cen C., Chen Z., Xu D., Jiang L., Chen X., Yi Z., Wu P., Li G., Yi Y. (2020). High quality factor, high sensitivity metamaterial graphene—Perfect absorber based on critical coupling theory and ımpedance matching. Nanomaterials.

[51] Masson J.F. (2020). Portable and field-deployed surface plasmon resonance and plasmonic sensor. Analyst.

[52] Zhan C., Liu B.W., Tian Z.Q., Ren B. (2020). Determining the interfacial refractive index via ultrasensitive plasmonic sensors. J. Am. Chem. Soc..

[53] Safran V., Göktürk I., Derazshamshir A., Yılmaz F., Sağlam N., Denizli A. (2019). Rapid sensing of Cu^+2^ in water and biological samples by sensitive molecularly imprinted based plasmonic biosensor. Microchem. J..

[54] Demirel G., Gieseking R.L.M., Ozdemir R., Kahmann S., Loi M.A., Schatz G.C., Facchetti A., Usta H. (2019). Molecular engineering of organic semiconductors enables noble metal-comparable SERS enhancement and sensitivity. Nat. Commun..

[55] Wang X., Yu S., Liu W., Fu L., Wang Y., Li J., Chen L. (2018). Molecular imprinting based hybrid ratiometric fluorescence sensor for the visual determination of bovine hemoglobin. ACS Sens..

[56] Schasfoort R.B.M. (2017). Chapter 3: Surface plasmon resonance instruments. Handbook of Surface Plasmon Resonance.

[57] Homola J. (2008). Surface plasmon resonance sensors for detection of chemical and biological species. Chem. Rev..

[58] Couture M., Zhao S.S., Masson J.F. (2013). Modern surface plasmon resonance for bioanalytics and biophysics. Phys. Chem. Chem. Phys..

[59] Mayer K.M., Hafner J.H. (2011). Localized surface plasmon resonance sensors. Chem. Rev..

[60] Inci F., Saylan Y., Kojouri A.M., Ogut M.G., Denizli A., Demirci U. (2020). A disposable microfluidic-integrated hand-held plasmonic platform for protein detection. Appl. Mater. Today.

[61] Liu L., Moore M.D. (2020). Survey of analytical techniques for noroviruses. Foods.

[62] Saylan Y., Erdem Ö., Cihangir N., Denizli A. (2019). Detecting fingerprints of waterborne bacteria on a sensor. Chemosensors.

[63] Luo L., Zhang F., Chen C., Cai C. (2019). Visual simultaneous detection of hepatitis A and B viruses based on a multifunctional molecularly imprinted fluorescence sensor. Anal. Chem..

[64] Monfared Y.E. (2020). Overview of recent advances in the design of plasmonic fiber-optic biosensors. Biosensors.

[65] Hassanpour S., Baradaran B., Hejazi M., Hasanzadeh M., Mokhtarzadeh A., de la Guardia M. (2018). Recent trends in rapid detection of influenza infections by bio and nanobiosensor. Trends Anal. Chem..

[66] Patil P.O., Pandey G.R., Patil A.G., Borse V.B., Deshmukh P.K., Patil D.R., Tade R.S., Nangare S.N., Khan Z.G., Patil A.M. (2019). Graphene-based nanocomposites for sensitivity enhancement of surface plasmon resonance sensor for biological and chemical sensing: A review. Biosens. Bioelectron..

[67] Ullah N., Mansha M., Khan I., Qurashi A. (2018). Nanomaterial-based optical chemical sensors for the detection of heavy metals in water: Recent advances and challenges. Trends Anal. Chem..

[68] Upadhyayula V.K.K. (2012). Functionalized gold nanoparticle supported sensory mechanisms applied in detection of chemical and biological threat agents: A review. Anal. Chim. Acta.

[69] Wang Y.F., Pan M.M., Yu X., Xu L. (2020). The recent advances of fluorescent sensors based on molecularly imprinted fluorescent nanoparticles for pharmaceutical analysis. Curr. Med. Sci..

[70] Kim J.B., Lee S.Y., Min N.G., Lee S.Y., Kim S.H. (2020). Plasmonic janus microspheres created from pickering emulsion drops. Adv. Mater..

[71] Redding B., Schwab M.J., Pan Y.L. (2015). Raman spectroscopy of optically trapped single biological micro-particles. Sensors.

[72] Nikoleli G.P., Nikolelis D.P., Tzamtzis N. (2012). Portable biosensors for the rapid detection of biochemical weapons of terrorism. Portable Chemical Sensors Weapons Against Bioterrorism.

[73] Dembek Z.P., Pavlin J.A., Siwek M., Kortepeter M.G. (2018). Epidemiology of biowarfare and bioterrorism. Medical Aspects of Biological Warfare.

[74] Primmerman C.A. (2000). Detection of biological agents. Linc. Lab. J..

[75] Bogomolova A. (2010). Sensing of biowarfare agents. Sensors for Chemical and Biological Applications.

[76] Carus W.S. (2015). The history of biological weapons use: What we know and what we don’t. Health Secur..

[77] Marston H.D., Folkers G.K., Morens D.M., Fauci A.S. (2014). Emerging viral diseases: Confronting threats with new technologies. Sci. Transl. Med..

[78] Centers for Diseases Control and Prevention (CDC) Bioterrorism Agents/Diseases. http://www.bt.cdc.gov/agent/agentlist-category.asp.

[79] Sharma P.K., Kumar J.S., Singh V.V., Biswas U., Sarkar S.S., Alam S.I., Dash P.K., Boopathi M., Ganesan K., Jain R. (2020). Surface plasmon resonance sensing of Ebola virus: A biological threat. Anal. Bioanal. Chem..

[80] Sikarwar B., Singh V.V., Sharma P.K., Kumar A., Thavaselvam D., Boopathi M., Singh B., Jaiswal Y.K. (2017). DNA-probe-target interaction based detection of *Brucella melitensis* by using surface plasmon resonance. Biosens. Bioelectron..

[81] Patel K., Halevi S., Melman P., Schwartz J., Cai S., Singh B.R. (2017). A novel surface plasmon resonance biosensor for the rapid detection of botulinum neurotoxins. Biosensors.

[82] Versiani A.F., Martins E.M.N., Andrade L.M., Cox L., Pereira G.C., Barbosa-Stancioli E.F., Nogueira M.L., Ladeira L.O., da Fonseca F.G. (2020). Nanosensors based on LSPR are able to serologically differentiate dengue from Zika infections. Sci. Rep..

[83] Dab C., Thomas R., Ruediger A. (2020). Design of a plasmonic platform to improve the SERS sensitivity for molecular detection. Photonic Sens..

[84] Mosier-Boss P.A. (2017). Review of SERS substrates for chemical sensing. Nanomaterials.

[85] Prakash O., Sil S., Verma T., Umapathy S. (2020). Direct detection of bacteria using positively charged ag/au bimetallic nanoparticles: A label-free surface-enhanced raman scattering study coupled with multivariate analysis. J. Phys. Chem. C.

[86] Wang R., Kim K., Choi N., Wang X., Lee J., HoJeon J., Rhie G.H., Choo R. (2018). Highly sensitive detection of high-risk bacterial pathogens using SERS-based lateral flow assay strips. Sens. Actuators B Chem..

[87] Yue G., Su S., Li N., Shuai M., Lai X., Astruc D., Zhao P. (2016). Gold nanoparticles as sensors in the colorimetric and fluorescence detection of chemical warfare agents. Coord. Chem. Rev..

[88] Smith R.G., D’Souza N., Nicklin S. (2008). A review of biosensors and biologically inspired systems for explosives detection. Analyst.

[89] Zhang W.Y., Guo Z.Z., Chen Y., Cao Y.P. (2017). Nanomaterial based biosensors for detection of biomarkers of exposure to op pesticides and nerve agents: A review. Electroanalysis.

[90] Chen L.Y., Wu D., Yoon J. (2018). Recent advances in the development of chromophore-based chemosensors for nerve agents and phosgene. ACS Sens..

[91] Saylan Y., Akgönüllü S., Çimen D., Derazshamshir A., Bereli N., Yılmaz F., Denizli A. (2017). Development of surface plasmon resonance sensors based on molecularly imprinted nanofilms for sensitive and selective detection of pesticides. Sens. Actuators B Chem..

[92] Akgönüllü S., Yavuz H., Denizli A. (2020). SPR nanosensor based on molecularly imprinted polymer film with gold nanoparticles for sensitive detection of aflatoxin B1. Talanta.

[93] Song Y., Bai J., Zhang R., Wu E., Wang J., Li S., Ning B., Wang M., Gao X., Peng Y. (2020). LSPR-enhanced photonic crystal allows ultrasensitive and label-free detection of hazardous chemicals. Sens. Actuators B Chem..

[94] Verma M.S., Chandra M. (2020). Nonlinear plasmonic sensing for label-free and selective detection of mercury at picomolar level. ACS Sens..

[95] Amirjani A., Haghshenas A.F. (2018). Ag nanostructures as the surface plasmon resonance (SPR)˗based sensors: A mechanistic study with an emphasis on heavy metallic ions detection. Sens. Actuators B Chem..

[96] Thenmozhi H., Rajana M.S.M., Ahmed K. (2019). D-shaped PCF sensor based on SPR for the detection of carcinogenic agents in food and cosmetics. Optik.

[97] Heleg-Shabtai V., Sharabi H., Zaltsman A., Ron I., Pevzner A. (2020). Surface-enhanced Raman spectroscopy (SERS) for detection of VX and HD in the gas phase using a hand-held Raman spectrometer. Analyst.

[98] Lafuente M., Pellejero I., Sebastián V., Urbiztondo M.A., Mallada R., Pina M.P., Santamaría J. (2018). Highly sensitive SERS quantification of organophosphorous chemical warfare agents: A major step towards the real time sensing in the gas phase. Sens. Actuators B Chem..

